# Disseminated extranodal NK/T-cell lymphoma presenting with bilateral adrenal masses: a case report and literature review

**DOI:** 10.3389/fonc.2026.1738153

**Published:** 2026-05-19

**Authors:** Pinyao Liang, Junxiong Li, Yumin Wang, Jingbo Qin, Ruwen Wang, Haotan Li, Yingxia Wang, Xiaodong Liu

**Affiliations:** 1 Second Department of Urology, The First Affiliated Hospital of Kunming Medical University, Kunming, Yunnan, China; 2 Department of Pathology, The First Affiliated Hospital of Kunming Medical University, Kunming, Yunnan, China

**Keywords:** adrenal neoplasms, chemotherapy, diagnosis, disseminated extranodal NK/T-cell lymphoma, Epstein-Barr virus

## Abstract

**Objective:**

To report a rare case of disseminated extranodal NK/T-cell lymphoma initially presenting with massive bilateral adrenal masses, and to discuss its clinical characteristics, diagnosis, and management based on the case and literature.

**Methods:**

A retrospective analysis was performed on the clinical data of a patient admitted to the Department of Urology, The First Affiliated Hospital of Kunming Medical University in June 2023.

**Results:**

A 58-year-old female patient presented with right flank and abdominal pain. Imaging revealed massive bilateral adrenal masses. Pathological diagnosis of NK/T-cell lymphoma was confirmed by biopsy, and further staging identified disseminated ENKTCL, stage IV, group A (high-risk). The patient received three cycles of GemOX chemotherapy (gemcitabine + oxaliplatin) but experienced rapid disease progression and died within three months of diagnosis.

**Conclusion:**

Extranodal NK/T-cell lymphoma can rarely present initially with bilateral adrenal masses. Its onset is insidious, and the prognosis is extremely poor. When encountering bilateral adrenal masses with atypical imaging features, lymphoma should be considered. Early biopsy to avoid unnecessary surgery, coupled with prompt initiation of systemic treatment through multidisciplinary collaboration, is crucial for improving outcomes in such patients.

## 1 Introduction

Extranodal NK/T-cell lymphoma (ENKTCL) is an aggressive Epstein-Barr virus (EBV)-associated non-Hodgkin lymphoma with a very poor prognosis (1–3). While its most common primary site is the upper aerodigestive tract (UAT), particularly the nasal cavity, and thus it is often referred to as nasal-type NK/T-cell lymphoma (4, 5). However, ENKTCL can also originate from extranodal sites outside the UAT (NUAT-ENKTCL), involving locations such as the skin, gastrointestinal tract, and testes. When ENKTCL involves the adrenal glands, it usually part of a disseminated (systemic) disease. Disseminated cases presenting with massive bilateral adrenal masses as the primary and dominant clinical manifestation is exceptionally rare. This mode of onset is insidious and can be easily confused on imaging with more common primary or metastatic adrenal tumors, frequently leading to diagnostic delay. The Department of Urology at The First Affiliated Hospital of Kunming Medical University admitted one such patient with disseminated ENKTCL involving both adrenal glands in June 2023. By reporting this case and reviewing the relevant literature, this article aims to: (1) summarize its atypical clinical and imaging features to aid differential diagnosis for urologists and radiologists; (2) discuss the diagnostic-therapeutic pathway, emphasizing the crucial role of multidisciplinary collaboration and precise pathological diagnosis in avoiding unnecessary surgery; and (3) analyze factors associated with its poor outcome and reflect on treatment strategies based on molecular characteristics. This study seeks to enhance clinical awareness of this rare presentation and provide a reference for early diagnosis and standardized management.

## 2 Clinical data

### 2.1 General information

The patient was a 58-year-old female admitted on June 30, 2023, due to “bilateral adrenal masses discovered on imaging one day prior.” She presented with right flank and abdominal pain without an obvious cause. A CT scan at the referring hospital indicated bilateral adrenal space-occupying lesions. She was admitted to our hospital for further evaluation. Her blood pressure upon admission was 109/77 mmHg. During the course of her illness, she experienced occasional dizziness and nausea, but no urinary frequency, urgency, dysuria, gross hematuria, palpitations, or sweating. There were no other symptoms such as chills or fever. Her past health was good, and physical examination revealed no significant abnormalities. There was no notable recent change in body weight.

### 2.2 Auxiliary examinations and pathology

Complete Blood Count: White Blood Cells 2.16×10^9^/L, Absolute Neutrophil Count 0.90×10^9^/L, Absolute Lymphocyte Count 1.00×10^9^/L, Mean Corpuscular Hemoglobin 26.90 pg, Red Cell Distribution Width-Standard Deviation 40 fL, Plateletcrit 0.18%.

Endocrine Function Tests: Afternoon (4:00 PM) plasma ACTH: 1.84 pg/mL (reference range 3–30 pg/mL), significantly below the lower limit of normal; Midnight (0:00) serum cortisol: 89.21 µg/dL (reference range <50 µg/dL).

Urinary System Ultrasound: Revealed solid masses in both adrenal regions. The right mass measured approximately 11.5cm × 7.8cm, and the left measured approximately 9.5cm × 6.7cm; nature pending investigation.

**Figure 1:**
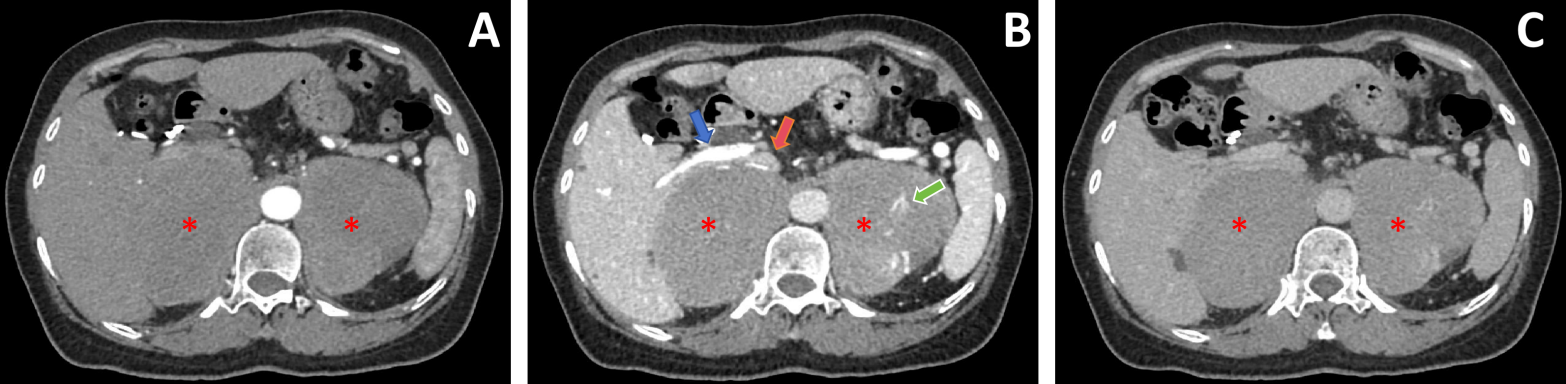
Dynamic contrast-enhanced CT images of bilateral adrenal masses. **(A)** Arterial phase: Large soft-tissue masses (asterisks) are present in the bilateral adrenal regions. **(B)** Venous phase: Large soft-tissue masses (asterisks) are present in the bilateral adrenal regions. This phase clearly demonstrates vessels traversing within and around the masses (green arrows), with compression and anterior displacement of the inferior vena cava (red arrow) and right renal vein (blue arrow). **(C)** Delayed phase: Large soft-tissue masses (asterisks) are present in the bilateral adrenal regions.

Adrenal Contrast-Enhanced CT: Showed symmetrical soft tissue masses in both adrenal regions. The right mass measured approximately 7.8cm × 8.3cm × 9.5cm, and the left measured approximately 7.2cm × 8.0cm × 8.6cm. Enhancement showed progressive mild enhancement. Vessels were seen traversing within and around the masses. The inferior vena cava and right renal vein were compressed and displaced anteriorly (**Figure 1**). The CT diagnosis suggested bilateral adrenal masses, likely neoplastic, with lymphoma among the differentials.

Biopsy: After excluding contraindications, the patient underwent an ultrasound-guided percutaneous adrenal needle biopsy under local anesthesia. Six cores were obtained with satisfactory sampling.

**Figure 2:**
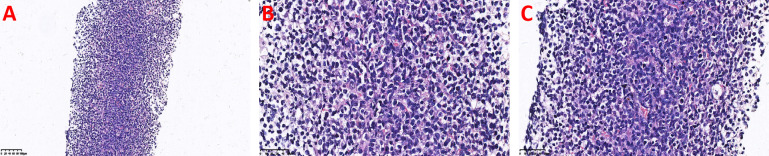
Histopathological examination of the adrenal biopsy specimen (H&E staining): **(A)** Low-power view shows effacement of the normal adrenal architecture by a diffuse infiltrate of tumor cells exhibiting an angiocentric growth pattern. **(B)** High-power view reveals scattered apoptotic bodies, necrotic cell debris, and karyorrhexis. The large cells have round nuclei with vacuolated or granular chromatin. **(C)** High-power view shows tumor cells of varying sizes, with irregular nuclear membranes, densely distributed chromatin, inconspicuous nucleoli, and pale-staining, scant to moderate cytoplasm.

Pathology:

H&E Staining: Showed effacement of the normal adrenal architecture replaced by a diffuse infiltrate of tumor cells exhibiting an angiocentric growth pattern and marked variation in size. Small cells featured irregular nuclear contours, densely packed chromatin, inconspicuous nucleoli, and pale-staining, scant to moderate cytoplasm; medium-sized to large cells displayed round nuclei with vacuolated to granular chromatin, pale to clear cytoplasm, and discernible nucleoli. Scattered apoptotic bodies, necrotic cell debris, and karyorrhexis were present (**Figure 2**).

**Figure 3:**
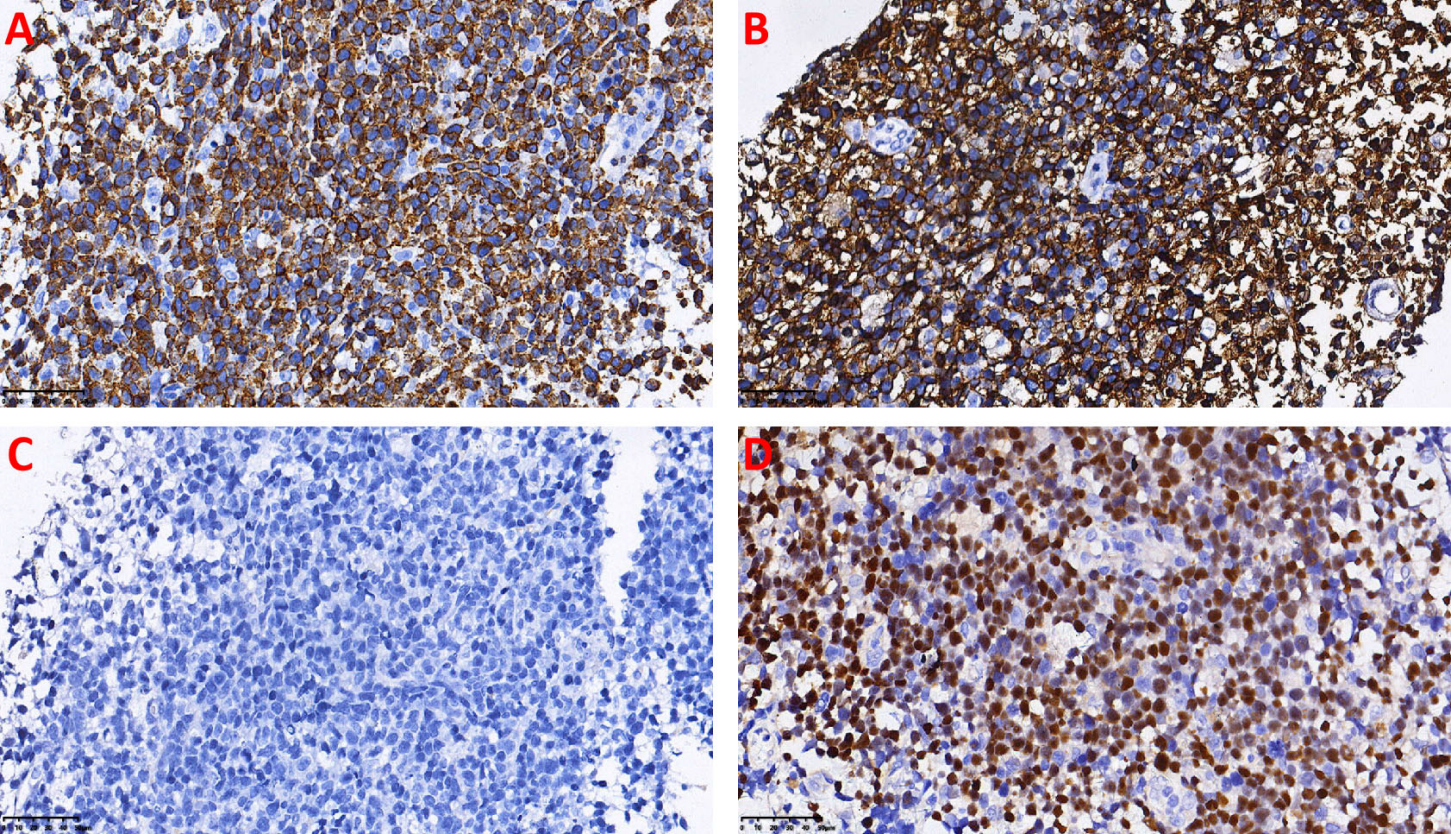
Immunohistochemistry and EBER *in situ* hybridization detection on the adrenal biopsy specimen: **(A)** Immunohistochemistry shows strong positivity for CD3 in the cytoplasm and membrane of the tumor cells. **(B)** Immunohistochemistry shows strong membranous positivity for CD56 in the tumor cells. **(C)** Immunohistochemistry shows negativity for CD20 in the tumor cells. **(D)** EBER *in situ* hybridization shows positive nuclear signals in the tumor cells.

Immunohistochemistry: CD3(+), CD20(-), CD79a(-), PAX5(-), CD10(-), MUM1(+), CD2(+), BCL6(-), BCL2(-), C-myc(+, 10%), CD5(-), CyclinD1(-), CD30(-), CD7(-), P53(-), Ki-67(+, 80%), CD4(+), CD8(-), CD21(-), CD56(+), GrB(+), TIA-1(+), TdT(-) (**Figure 3**).

*In Situ* Hybridization: EBER(+).

Pathological Diagnosis: NK/T-cell lymphoma.

### 2.3 Hematology workup and final diagnosis

The patient recovered well post-procedure, was discharged smoothly, and transferred to the Department of Hematology for further management. Relevant investigations were completed:

Laboratory Tests: Revealed significantly elevated tumor and infection markers: Lactate Dehydrogenase (798 IU/L), β2-Microglobulin (4.98 mg/L), and Ferritin (1396.1 μg/L). EBV-DNA quantification was high at 2.0 × 10^4^ copies/ml.

Imaging:

Superficial Lymph Node Ultrasound: Revealed multiple lymph nodes, some enlarged.

Chest CT: Showed few tiny nodules in both lungs, bilateral pulmonary bullae, and small bilateral pleural effusions.

PET-CT: Showed: 1. Large masses in both adrenal regions, a right breast nodule, right maxillary sinus involvement, and multiple lymph nodes in the mediastinum and abdomen, all with increased metabolism, suggesting lymphoma infiltration. 2. Scattered inflammatory foci in the lungs, inflammation in the right ethmoid sinus, left maxillary sinus, and bilateral palatine tonsils, possibly inflammatory/tumor infiltration.

**Figure 4:**
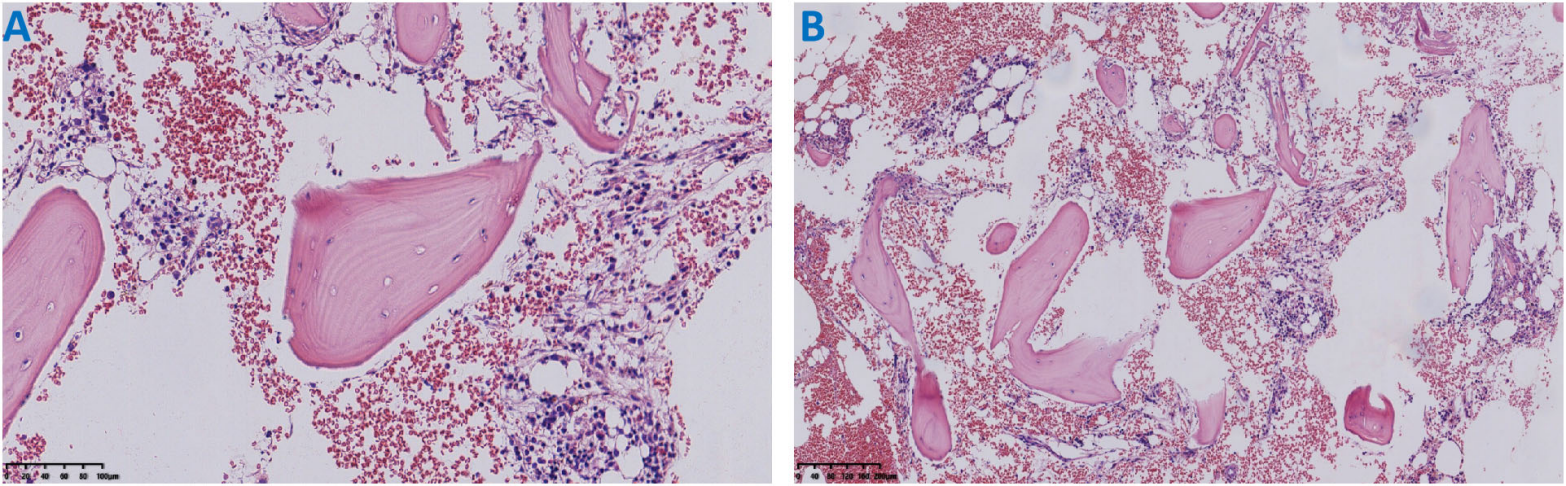
Histopathological examination of the bone marrow biopsy specimen (H&E staining): **(A)** The bone marrow tissue shows trilineage hematopoiesis (granulocytic, erythroid, and megakaryocytic series). All cell lineages exhibit roughly normal differentiation and morphology, with no definite evidence of tumor cell infiltration. **(B)** Focal bone marrow tissue with accompanying hemorrhage is observed.

Bone Marrow Examination: (Including bone marrow aspiration cytology, flow cytometry immunophenotyping, leukemia fusion gene testing, and bone marrow biopsy pathology) showed no evidence of tumor involvement (**Figure 4**).

Final Diagnosis: Disseminated Extranodal NK/T-cell Lymphoma Stage IV (Group A, High-risk).

### 2.4 Treatment and outcome

Considering the primary adrenal presentation and high risk of central nervous system (CNS) involvement, a lumbar puncture was performed; cerebrospinal fluid analysis showed no malignant cells. Intrathecal chemotherapy (Methotrexate 10mg, Cytarabine 50mg, Dexamethasone 5mg) was administered slowly into the subarachnoid space for CNS lymphoma prophylaxis. Subsequently, the patient received the GemOX regimen (Oxaliplatin 155mg d1 + Gemcitabine 1.55g d1) chemotherapy, along with supportive care including antiemetics, gastric acid suppression, liver protection, diuretics, potassium supplementation, and urine alkalinization. During chemotherapy, the patient experienced adverse effects such as myelosuppression, nausea, and vomiting, which improved with granulocyte colony-stimulating factor and symptomatic treatment. Before the third cycle of chemotherapy on September 5, 2023, the EBV-DNA quantification was 4.5 × 10^2^ copies/mL, showing a significant decrease from previous levels. However, after discharge following this cycle, her condition deteriorated rapidly, and she unfortunately passed away.

## 3 Discussion

Extranodal NK/T-cell lymphoma (ENKTCL), is an aggressive non-Hodgkin lymphoma closely associated with Epstein-Barr virus (EBV) infection (1–3). It has also been termed angiocentric T-cell lymphoma and lethal midline granuloma, among other names. NK/T-cell lymphoma (NK/TCL) was formally named in the Revised European-American Lymphoma (REAL) Classification proposed in 1994 (6). This classification framework was subsequently adopted in the first edition of the World Health Organization (WHO) classification of tumors of haematopoietic and lymphoid tissues, published in 2000. In the 2001 edition of the WHO classification, “ENKTCL” was established as an independent pathological subtype (7). By 2016, ENKTCL was further defined as the most common EBV-positive T and NK-cell lymphoma (8). This disease is more prevalent in Asian and Central/South American populations (9, 10), with a male predominance noted in Asian cohorts (11, 12). ENKTCL can be classified based on the primary site into Upper Aerodigestive Tract (UAT)-NKTCL and Non-Upper Aerodigestive Tract (NUAT)-NKTCL. UAT-NKTCL primarily involves sites like the nasal cavity. When it originates from extranodal sites outside the UAT, such as the skin, gastrointestinal tract, or testis, it is typically classified as NUAT-NKTCL. Although relatively less common, NUAT-NKTCL tends to be more aggressive, and patients often have a poorer prognosis (4, 5).

The classic clinical presentation of ENKTCL centers on the UAT, often manifesting as refractory ulcers with extensive necrosis in the midfacial region (13), and initial symptoms such as nasal obstruction, abnormal nasal discharge, and epistaxis (8). The disease has a characteristic immunophenotype, typically showing cytoplasmic CD3ϵ(+), CD56(+), CD5(-), positive cytotoxic markers (e.g., GrB, TIA-1), and positive EBER *in situ* hybridization (14–16). However, when the tumor originates outside the UAT, its clinical presentation is non-specific and can vary greatly depending on the involved organ. For instance, gastrointestinal involvement may present with abdominal pain, bleeding, or perforation (17), while primary adrenal involvement often presents as an abdominal mass and related compressive symptoms. The case reported here is an extremely rare instance of ENKTCL presenting primarily and predominantly with massive bilateral adrenal masses. The patient sought medical attention for right flank and abdominal pain, and imaging revealed large space-occupying lesions in both adrenal regions. This clinical picture aligns with findings from Liu et al. (11), whose study indicated that NUAT-NKTCL patients not only present at a more advanced clinical stage but also have a significantly lower 5-year overall survival rate (34.7%) compared to UAT-NKTCL patients (64.2%), suggesting a worse prognosis for the former. Diagnosis in this case was confirmed by biopsy. H&E staining showed replacement of normal adrenal structure by a diffuse infiltrate of markedly atypical tumor cells with characteristic angiocentric growth, accompanied by apoptotic bodies and necrosis, highly suggestive of an aggressive lymphoma. Further confirmation by immunohistochemistry and molecular testing revealed that the tumor cells expressed CD56, GrB, and TIA-1, and EBER *in situ* hybridization was positive. These morphological and immunophenotypic features corroborated each other, collectively indicating a tumor derived from the NK/T-cell lineage with highly aggressive biological behavior.

Furthermore, high serum EBV-DNA copy numbers are closely associated with tumor burden and poor survival prognosis (18). The clinical features and key molecular biological indicators in this case were highly consistent: the EBV-DNA quantification was as high as 2.0 × 10^4^ copies/ml, providing strong molecular evidence of a massive tumor burden. Literature reports indicate that adrenal involvement in ENKTCL often presents radiologically as bilateral involvement, clinically often with abdominal or back pain as the initial symptom, and carries a worse prognosis when multiple sites are involved (7), consistent with this case. It is noteworthy that according to the Ann Arbor-Cotswolds staging system, lymphoma can be categorized into Group A or B based on the presence of systemic symptoms. B symptoms include unexplained fever, night sweats, and weight loss >10% within six months (19). Although this patient had an extremely high tumor burden and was Stage IV, she was clinically assessed as Group A, meaning she lacked the aforementioned B symptoms. This contrast between the massive tumor burden and the relatively “quiet” systemic clinical presentation profoundly illustrates that ENKTCL, particularly when involving deep-seated organs like the adrenal glands, can have an extremely insidious onset, lacking typical systemic warning signs, thereby more easily leading to delayed diagnosis. Although the patient had involvement of additional extranodal sites such as the maxillary sinus and mammary lymph nodes, confirming its disseminated nature, the clinical presentation—dominated by the massive adrenal-centered tumor burden, extremely high EBV-DNA levels, and rapid disease progression—more accurately aligns with the profile of disseminated non-upper aerodigestive tract NK/T-cell lymphoma (NUAT-ENKTCL) with primary adrenal manifestation. This situation indicates that for advanced cases with multiple extranodal involvements, clinical subtyping based on the principle of dominant tumor burden, combined with the objective indicator of EBV-DNA, is crucial for accurately assessing their extremely aggressive prognosis.

From the perspective of urological clinical practice, this case provides important insights into the diagnosis of rare lymphomas presenting primarily as bilateral adrenal masses. Firstly, endocrine evaluation suggested the tumors were non-functional or associated with mild hormonal rhythm disturbances. Building on this, imaging differential diagnosis provided key clues: the contrast-enhanced CT in this case showed large, symmetrical soft tissue masses in the adrenal regions. The pattern of “progressive mild enhancement” differed significantly from the “wash-in/wash-out” pattern typical of highly vascular pheochromocytoma or the heterogeneous marked enhancement of adrenocortical carcinoma. More characteristically, the imaging showed anterior displacement of the inferior vena cava and right renal vein due to compression, with vessels traversing within and around the masses, indicating generally preserved vascular morphology without clear evidence of invasion or truncation. This reflects a “pushing” rather than “infiltrative-destructive” growth pattern of lymphoma, distinct from the vascular invasion and encasement often seen in carcinomas. Additionally, despite the large tumor volume, the internal density was relatively homogeneous, without clear non-enhancing necrotic areas, a feature that also helps differentiate it from adrenocortical carcinoma or metastases which are prone to necrosis.

The rapid death of the patient following diagnosis highlights a fundamental dilemma in the management of late-stage NUAT-ENKTCL presenting with adrenal involvement: the tension between diagnostic complexity and the narrow window for effective treatment. This compels a deeper reflection on our clinical decision-making logic. First, optimizing the diagnostic-therapeutic pathway is a prerequisite for effective management. The success in this case lies in the clinical team’s decision to perform a percutaneous biopsy rather than proceeding directly to high-risk surgical resection upon discovering the atypical adrenal mass. This approach avoided unnecessary surgical trauma and gained critical time for transitioning the patient to systemic medical therapy, underscoring the principle that precise diagnosis must precede therapeutic intervention. For suspected cases of this rare lymphoma, establishing a rapid pathway from imaging screening to minimally invasive biopsy and pathological confirmation represents the first step toward improving outcomes. Second, multidisciplinary team (MDT) collaboration should run through the entire diagnostic-treatment process, with its core being the early timing of decision-making. In this case, the sequential cooperation among urology, radiology, pathology, and hematology departments formed the foundation for correct management. However, a more ideal model would involve early MDT engagement upon the initial detection of a complex adrenal mass, jointly formulating an integrated strategy that includes differential diagnosis, biopsy planning, staging work-up, and treatment direction—rather than simple referral between specialties at different stages. Such in-depth collaboration can minimize diagnostic delays and ensure that treatment decisions are both scientifically sound and efficiently implemented. Finally, the outcome of this case prompts reflection on the limitations of current treatment strategies and future directions. Despite the patient receiving standard chemotherapy and central nervous system prophylaxis, the disease remained uncontrollable, reflecting the limitations of conventional therapies for some extremely high-risk NUAT-ENKTCL cases. The molecular features of this case suggest that for similar patients in the future, evaluating the feasibility of enrolling in clinical trials for novel therapies—such as immune checkpoint inhibitors or targeted agents—early after diagnosis may represent an important avenue for achieving therapeutic breakthroughs.

## 4 Conclusion

In summary, although ENKTCL can rarely present initially with bilateral adrenal masses, its onset is insidious, progression rapid, and prognosis extremely poor. This case suggests that when encountering patients presenting with large bilateral adrenal masses showing “progressive mild enhancement” on imaging and hormone levels not supporting common functional tumors, urologists should include ENKTCL high in the differential diagnosis. For such atypically featured masses, especially after excluding pheochromocytoma, actively performing a biopsy should be a key diagnostic step. This helps avoid unnecessary surgical resection and secures valuable time for patients to transition to medical oncology management. Therefore, placing biopsy at the core of the diagnostic workflow is essential for achieving early diagnosis and formulating the correct treatment plan. This case offers dual insights for clinical practice: On the diagnostic level, when encountering patients with large bilateral adrenal masses showing “progressive mild enhancement” on imaging yet lacking hormonal profiles typical of common functional tumors, urologists should include ENKTCL among the primary differential diagnoses. Performing an early biopsy is a key step to avoid unnecessary surgery and achieve timely pathological confirmation. On the management level, the care of such high-risk patients must rely on early and deeply integrated multidisciplinary collaboration (MDT). From the initial imaging assessment and biopsy decision to pathological diagnosis and systemic treatment, the MDT should be involved throughout to develop an efficient, unified strategy. At the same time, it is essential to recognize the limitations of conventional chemotherapy for some extremely high-risk patients. Based on molecular characteristics, we should actively explore individualized, cutting-edge strategies incorporating immunotherapy and targeted agents, while seeking opportunities for patients to participate in clinical trials. Therefore, building a diagnostic-therapeutic model centered on precise and rapid diagnosis, supported by the dual pillars of multidisciplinary collaboration and individualized treatment strategies, is crucial for improving outcomes in such rare and aggressive cases.

## Data Availability

The original contributions presented in the study are included in the article/supplementary material. Further inquiries can be directed to the corresponding authors.

## References

[1] Al-Naeeb AB Ajithkumar T Behan S Hodson DJ . Non-hodgkin lymphoma. Bmj. (2018) 362. doi: 10.1136/bmj.k3204. PMID: 30135071

[2] Cai Q Cai J Fang Y Young KH . Epstein-Barr virus-positive natural killer/T-cell lymphoma. Front Oncol. (2019) 9:386. doi: 10.3389/fonc.2019.00386. PMID: 31139570 PMC6527808

[3] Qi SN Yang Y Zhang YJ Huang HQ Wang Y He X . Risk‐based, response‐adapted therapy for early‐stage extranodal nasal‐type NK/T‐cell lymphoma in the modern chemotherapy era: a China Lymphoma Collaborative Group study. Am J Hematol. (2020) 95:1047–56. doi: 10.1002/ajh.25878 32449800

[4] Liu ZL Bi XW Zhang XW Lei DX Liu PP Yang H . Characteristics, prognostic factors, and survival of patients with NK/T-cell lymphoma of non-upper aerodigestive tract: a 17-year single-center experience. Cancer Res Treat Off J Korean Cancer Assoc. (2019) 51:1557–67. doi: 10.4143/crt.2018.681. PMID: 30971067 PMC6790852

[5] Wang MH Luo ZF Chen MJ Wu WT Zheng YF Yuan ZY . Analysis of clinicopathological features of 24 young patients with extranodal NK/T-cell lymphoma, nasal type [in Chinese. Chin J Clin Exp Pathol. (2018) 34:337–40.

[6] Harris NL . American classification of lymphoid neoplasms; a proposal from the International Lymphoma Study Group. Blood. (1994) 84:1361–92. doi: 10.1111/j.1365-2559.1994.tb01371.x. PMID: 8068936

[7] Jaffe ES . World Health Organization classification of tumors. Pathology and genetics of tumors of hematopoietic and lymphoid tissues. World Health Organ Classif Tumors Pathol Genet Tumors Hematopoietic Lymphoid Tissues. (2001) 3:185–7. doi: 10.1093/annonc/mdf146

[8] Swerdlow SH Campo E Pileri SA Harris NL Stein H Siebert R . The 2016 revision of the World Health Organization classification of lymphoid neoplasms. Blood. (2016) 127:2375–90. doi: 10.1182/blood-2016-01-643569. PMID: 26980727 PMC4874220

[9] Ji XY Shen DP Yang YQ Wei YF Huang X Liu Q . Clinical analysis of primary adrenal NK/T-cell lymphoma [in Chinese. J Exp Hematol. (2023) 31:396–402. doi: 10.19746/j.cnki.issn.1009-2137.2023.02.013 37096511

[10] Cai J Cao Y Qiu LY Gao Y Huang HQ Cai QQ . Research progress on EBV-associated NK/T-cell lymphoma [in Chinese. Sci Sin Vitae. (2024) 54:2363–84.

[11] Jin JY Yu YR . NK/T cell lymphoma involving bilateral adrenal glands: a case report [in Chinese. Chin J Endocrinol Metab. (2005) 21:82.

[12] Lyu YM Wei YF Zhao LL Jin S Zhang L Tao L . Primary bilateral adrenal NK/T-cell lymphoma: a case report and literature review [in Chinese. Chin J Clin Exp Pathol. (2019) 35:974–6.

[13] Lu HF Sun MH Wang J Sheng WQ Xu YX Shi DR . Clinicopathological characteristics and immunophenotype of primary adrenal nasal type NK/T-cell lymphoma [in Chinese. China Oncol. (2003) 13:548–51.

[14] Chan JK . Natural killer cell neoplasms. Anat Pathol (Chicago Ill annual). (1998) 3:77–145. doi: 10.1142/s0219836304000500. PMID: 10389582

[15] Chan JK Tsang WY Ng CS . Clarification of CD3 immunoreactivity in nasal T/natural killer cell lymphomas: the neoplastic cells are often CD3ϵ+. Blood. (1996) 87:839–41. doi: 10.1182/blood.v87.2.839.bloodjournal872839 8555511

[16] Jaffe ES . Nasal and nasal‐type T/NK cell lymphoma: a unique form of lymphoma associated with the Epstein‐Barr virus. Histopathology. (1995) 27:581–3. doi: 10.1111/j.1365-2559.1995.tb00333.x. PMID: 8838342

[17] Kim SJ Jung HA Chuang SS Hong H Guo CC Cao J . Extranodal natural killer/T-cell lymphoma involving the gastrointestinal tract: analysis of clinical features and outcomes from the Asia Lymphoma Study Group. J Hematol Oncol. (2013) 6:86. doi: 10.1186/1756-8722-6-86. PMID: 24238138 PMC4225665

[18] Fei Q Tian XK Wu J Zhu HM Wang Y Peng FY . Prognostic significance of Epstein–Barr virus DNA in NK/T-cell lymphoma: a meta-analysis. Onco Targets Ther. (2018) 2018:997–1004. doi: 10.2147/ott.s153942. PMID: 29520150 PMC5833780

[19] Lister TA Crowther D Sutcliffe SB Glatstein E Canellos GP Young RC . Report of a committee convened to discuss the evaluation and staging of patients with Hodgkin's disease: Cotswolds meeting. J Clin Oncol. (1989) 7:1630–6. doi: 10.1200/jco.1989.7.11.1630. PMID: 2809679

